# A case of atypical reninoma with mild hypertension and normal plasma renin activity but elevated plasma renin concentration

**DOI:** 10.1186/s12902-022-00977-w

**Published:** 2022-03-18

**Authors:** Baoping Wang, Li Ding, Shuanghua Xu, Yuxin Fan, Jiabo Wang, Xin Zhao, Diansheng Fu, Bo Bian, Kunlong Tang, Chunsheng Ni, Zuoliang Dong, Qing He, Ming Liu

**Affiliations:** 1 Department of Endocrinology and Metabolism, Tianjin Medical University General Hospital, Tianjin, China; 2 Department of Clinical Medicine, Tianjin Medical University, Tianjin, China; 3 Department of Imaging, Tianjin Medical University General Hospital, Tianjin, China; 4 Department of Cardiology, Tianjin Medical University General Hospital, Tianjin, China; 5 Department of Urology, Tianjin Medical University General Hospital, Tianjin, China; 6 Department of Pathology, Tianjin Medical University General Hospital, Tianjin, China; 7 Department of Medical Laboratory, Tianjin Medical University General Hospital, Tianjin, China

**Keywords:** Reninoma, Atypical reninoma, Plasma renin concentration, Plasma renin activity, Case report

## Abstract

**Background:**

Reninoma is a rare, benign renal neoplasm. Typical clinical features include severe hypertension, secondary hyperaldosteronism, hypokalaemia and metabolic alkalosis caused by the overproduction of renin.

**Case presentation:**

A 25-year-old lean Chinese woman with no family history of hypertension was hospitalized for stage 1 hypertension that gradually developed over two years. Endocrine investigation showed hyperreninemia without hyperaldosteronism and hypokalaemia. Interestingly, although the patient had an elevated plasma renin concentration (PRC), her plasma renin activity (PRA) was in the normal range. Abdominal contrast-enhanced computed tomography (CT) scanning revealed a solid, low-density, renal cortical mass with delayed enhancement. Selective renal vein sampling (SRVS) was performed, and a lateralization of the renin secretion from the left kidney was found. Enucleation of the tumour led to a rapid remission of hypertension and hyperreninemia. Based on pathological findings, the patient was diagnosed with reninoma. Immunohistochemical staining of the tumour was positive for Renin, CD34, Vimentin, and synaptophysin (Syn) and negative for somatostatin receptor 2 (SSTR2) and chromogranin A (CgA).

**Conclusions:**

Reninoma can present as mild hypertension without hyperaldosteronism and hypokalaemia. The clinical features of reninoma may depend on the degree of activation of the renin-angiotensin-aldosterone system (RAAS). PRC should be incorporated in the differential diagnosis of secondary hypertension.

## Background

Reninoma, which arises from modified smooth muscle cells in the wall of the glomerular afferent arterioles that are part of the juxtaglomerular apparatus [1], was first reported by Robertson in 1967 [2]. Since then, approximately 170 cases have been reported in the literature, and reninoma can occur at any age but is more frequent in young females [3]; the peak age of incidence is in the second and third decades of life [4]. Most cases are benign neoplasms, with very few reports of metastasized tumours [1, 5, 6]. Reninoma typically shows clinical features secondary to the overproduction of renin, including hyperaldosteronemia, hypokalaemia, and metabolic alkalosis, in addition to hypertension [7]. In this article, we report a unique and enlightening case of a reninoma in a female with an atypical clinical presentation, in whom there was an elevated plasma renin concentration (PRC), not elevated plasma renin activity (PRA).

## Case prensentation

Two years before admission, a 25-year-old female presented with borderline elevated blood pressure of 140/90 mmHg, which progressively increased to 150/100 mmHg, and drew the patient to seek medical attention at a local hospital two weeks prior to this admission. Laboratory tests revealed normal plasma aldosterone concentration (PAC) and PRA (radioimmunoassay, Beijing North Institute of Biotechnology Co, Ltd, accuracy: relative deviation in the range of ±10.0%) in both supine and upright positions during the follicular phase of the menstrual cycle (Table 1). The patient was given amlodipine 2.5 mg qd, and her blood pressure was well controlled. She had no family history of hypertension or kidney tumours. Upon admission, amlodipine was discontinued to facilitate accurate evaluation of levels of renin and PAC, and diltiazem at 90 mg qd was used instead. Informed consent was obtained from the patient for use of all data and images in this case report.

**Table 1:** The values of PRC, PRA and PAC in the supine and upright positions

Order	Position	PRC (uIU/ml)	Normal range (uIU/ml)	PRA (ng/ml·h)	Normal range (ng/ml·h)	PAC (pg/dl)	Normal range (pg/dl)
1	Supine position			1.12	0.13-1.74	58.8	30-180
	Upright position			1.45	1.45-5.0	80.19	50-313
2	Supine position	219.2	2.8-39.9			22.5	3.0-23.6
	Upright position	437.2	4.4-46.1			32.3	3.0-35.3

*PRC* Plasma renin concentration, *PRA* Plasma renin activity, *PAC* Plasma aldosterone concentration

Physical examination showed a blood pressure of 133/96 mmHg in the right arm and 137/97 mmHg in the left arm in the supine position, with a regular pulse of 72 bpm. The patient’s height was 164 cm, and weight was 52 kg with a normal body mass index of 19.3 kg/m^2^. Facial rounding, plethora, development of supraclavicular fat pads, purple skin striae, hirsutism, goiter, proptosis, orthostatic hypotension, and abdominal bruits were not evident upon physical examination.

Biochemical assays showed normal liver and renal function, with no hypokalaemia (K 3.8 mmol/L) or metabolic alkalosis (total carbon dioxide 28 mmol/L). The PRC (chemiluminescence immunoassay, DiaSorin Ltd, accuracy: relative deviation in the range of ±15.0%) and PAC were first evaluated during the luteal phase of the menstrual cycle, and elevated PRCs of 248.3 μIU/ml (normal range, 2.8–39.9 μIU/ml) and >500 μIU/ml (normal range, 4.4-46.1 μIU/ml) were evident in both the supine and upright positions, respectively, with normal PAC (15.2 pg/dl in the supine position [normal range, 3.0-23.6 pg/dl] and elevated PAC of 90.8 pg/dl in the upright position [normal range, 3.0-35.3 pg/dl]). The PRC and PAC were re-evaluated during the follicular phase of the menstrual cycle with the same methods, and elevated PRC values of 219.2 μIU/ml and 437.2 μIU/ml were evident in both the supine and upright positions, respectively, with normal PAC values of 22.5 pg/dl in the supine position and 32.3 pg/dl in the upright position (Table 1). The catecholamine metabolites, growth hormone, thyroid hormone, gonadal hormone, adrenocorticotropic hormone (ACTH), cortisol, and prolactin levels were all within the normal range (Table 2).

**Table 2:** Laboratory findings of hormone levels

	Value	Normal range
ACTH	12.4	0-46 pg/ml
Serum cortisol	12.9	5-25 μg/dl
VMA	2.6	<72umol/24 h
FSH	2.7	2.5-10.2IU/L
LH	3.8	1.9-12.5IU/L
E2	174	19-144 pg/ml
T	33.5	14-76 ng/dl
PRL	23.3	2.8-29.2 ng/ml
FT3	5.57	3.5-5.5 ng/dl
FT4	15.51	11.5-23.5 ng/dl
TSH	1.44	0.3-5.0 uIU/ml

*ACTH* Adrenocortical hormone, *VMA* 24-hurinary vanillylmandelicacid, *FSH* Follicle-stimulating hormone, *LH* Luteinizing hormone, *E2* Oestradiol, *PRL* Prolactin, *T* Testosterone, *FT3* Free triiodothyronine, *FT4* Free thyroxin, *TSH* Thyroid stimulating hormone

**Fig. 1:**
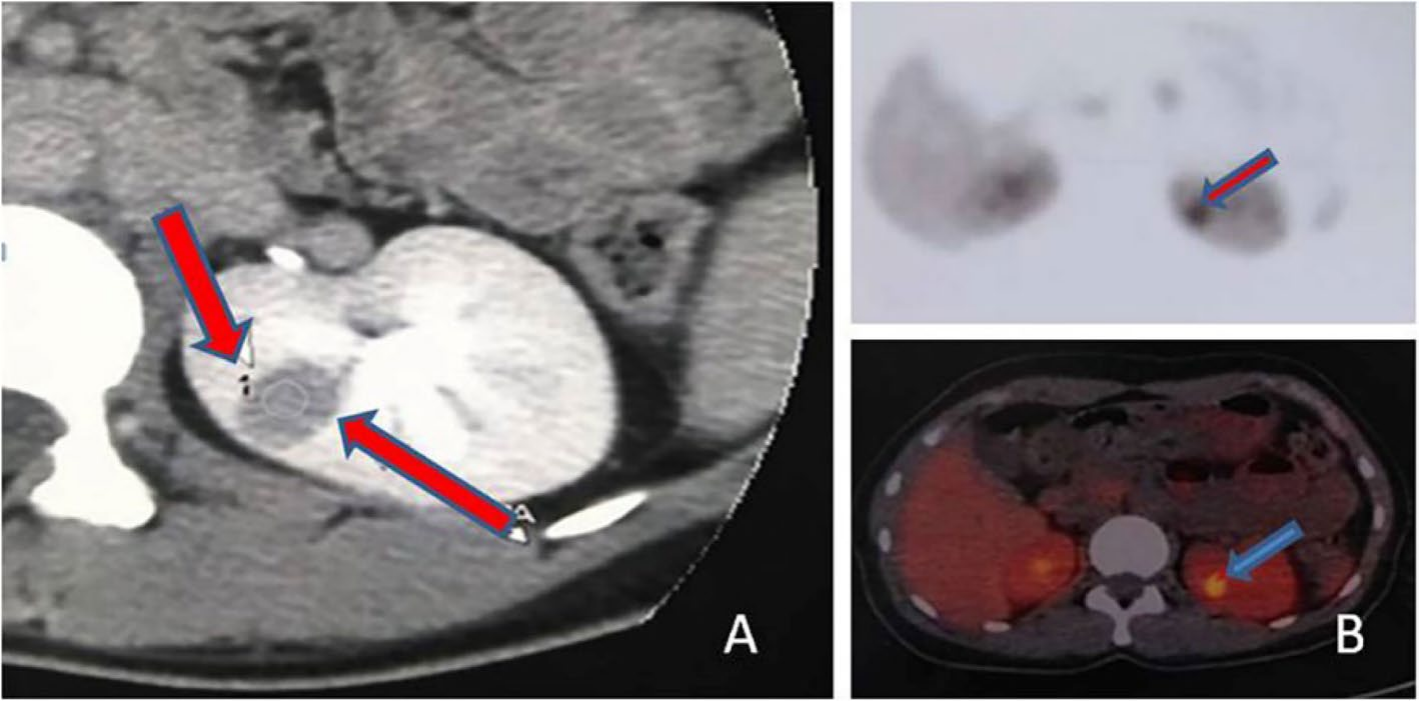
Abdominal contrast-enhanced CT and 18F-FDG PET-CT. **A** solid, circumscribed, low-density, cortical lesion (15 mm x13 mm) in the posterior lip of the left kidney, which appears to be moderately enhanced. There was no obvious enhancement in the arterial phase, but it was progressive in the venous phase and delayed phase. **B** mild FDG uptake in the mass

Abdominal contrast-enhanced CT scanning showed normal renal arteries, abdominal aorta and adrenal glands, with a solid, circumscribed, low-density, cortical lesion (15 mm x13 mm) in the posterior lip of the left kidney, which appeared to be moderately enhanced. There was no obvious enhancement in the arterial phase, but it was progressive in the venous phase and delayed phase (Fig. 1A). Magnetic resonance imaging (MRI) scanning plus contrast-enhanced MRI both confirmed this renal cortical lesion. 18F-Fluorodeoxyglucose (18F-FDG) PET-CT showed mild FDG uptake in the mass (Fig. 1B).

Reninoma was suspected, and selective renal vein sampling (SRVS) was performed. The renal vein renin ratio (RVRR) was 1.4, suggesting lateralization of the renin secretion to the left kidney (Table 3).

**Table 3:** The results of SRVS and RVRR

	PRC (uIU/ml)	Normal range (uIU/ml)	PAC (pg/dl)	Normal range (pg/dl)
Left renal vein	258.1	2.8-39.9	17	3.0-23.6
Right renal vein	183.2		17.4	
Proximal end of inferior vena cava	212.1		24.3	
Distal end of inferior vena cava	183.6		19.6	
RVRR of left/right side	1.4			

*SRVS* Selective renal vein sampling, *RVRR* Renal vein renin ratio

**Fig. 2:**
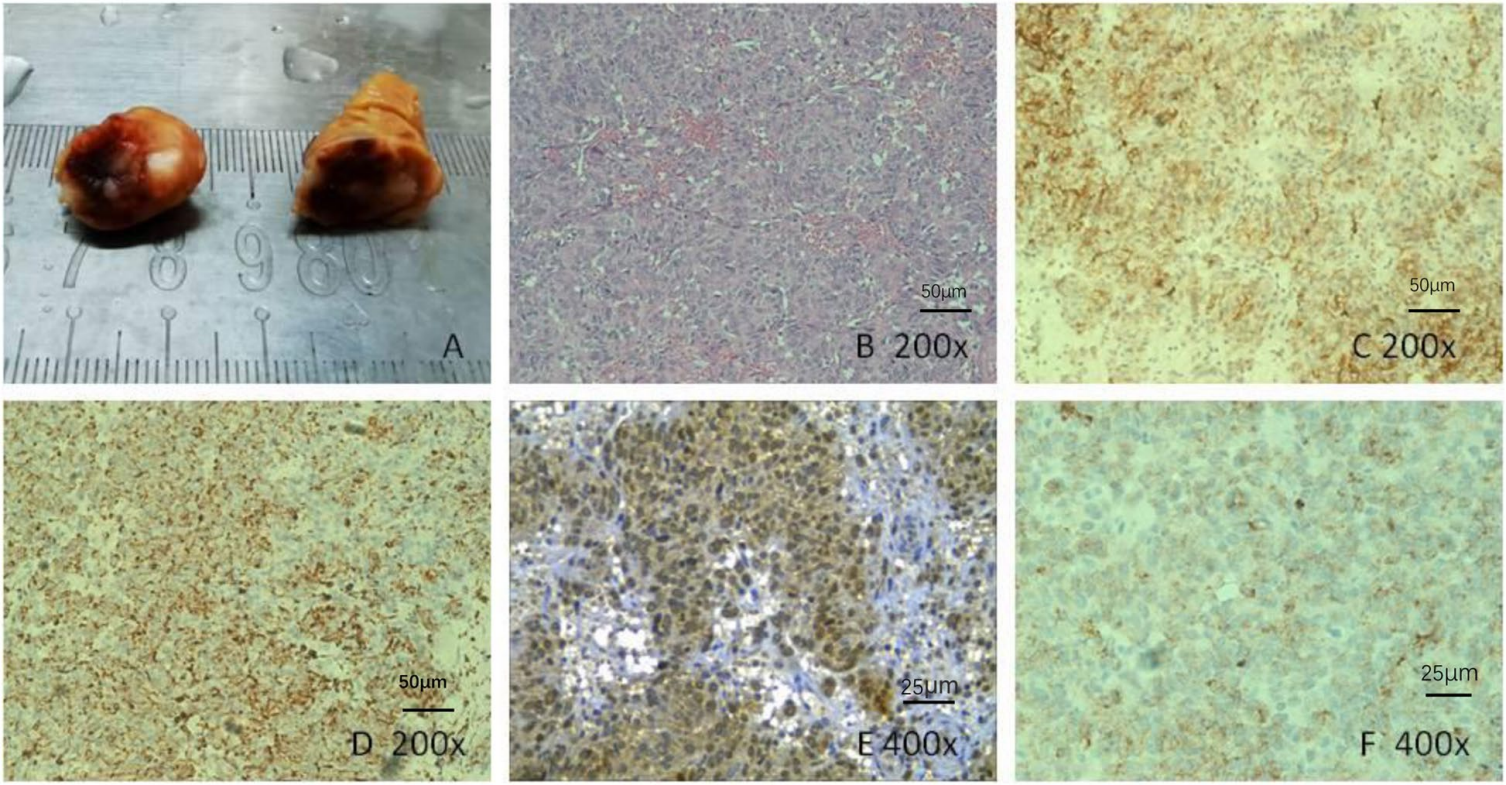
External appearance and histopathology of the reninoma. **A** External appearance of the tumour, 1 cm in diameter and tan in colour. **B** Histopathology revealed a neoplasm composed of solid sheets of closely packed polygonal cells with eosinophilic cytoplasm (HE staining 200×). Positive immunohistostaining is shown for CD34 (**C** 200×), vimentin (**D** 200×), renin (**E** 400×) and Syn (**F** 400×)

The patient underwent successful retroperitoneal laparoscopic nephron-sparing tumour enucleation and experienced uneventful recovery. The tumour, 1.5×1×1 cm, was well circumscribed and tan in colour (Fig. 2A). Haematoxylin-eosin staining revealed a neoplasm composed of solid sheets of closely packed polygonal cells with eosinophilic cytoplasm (Fig. 2B, Carl Zeiss confocal microscope Axio Imager M2 and the Zeiss ZEN microscope software, Oberkochen,Germany). Immunohistochemical staining showed positive staining for CD34 (Fig. 2C), vimentin (Fig. 2D), renin (Fig. 2E), and Syn (Fig. 2F), with a Ki-67 labelling index of approximately 5%. No positivity for CK7, CD10, 34βE12, EMA, HMB45, SMA, CgA, or SSTR2 immunostaining was noticed.

One day after surgery, the patient’s blood pressure normalized to 130/70 mmHg without antihypertensive drugs, and the PRC decreased from 219.2 μIU/ml to 9.8 μIU/ml. The patient remained normotensive at 6 months after surgery.

## Discussion and conclusion

Reninoma, a rare renal neoplasm and that is more frequently seen in young females, produces excessive amounts of renin, leading to secondary hyperaldosteronism and then hypertension and hypokalaemia [7]. In the current case, a reninoma was suggested by hypertension, hyperreninemia and the absence of artery stenosis in a young female with normal weight, no family history of hypertension and no drugs interfering with the PRC. The final diagnosis of reninoma was confirmed by positive immunostaining for renin, CD34, and vimentin in tumour cells, which is consistent with previous reports [8].

Interestingly, the patient had elevated PRC, but PRA was in the normal range. To date, approximately 170 cases have been reported, and either elevated PRA [3, 8–10] or elevated PRC [11, 12] is recorded in the literature. PRA, which reflects the biological activity of the renin system, is measured by generating angiotensin I (AngI) from endogenous angiotensinogen, followed by measurement by radioimmunoassay of the generated AngI. The assay may not necessarily reflect the real concentration of active renin, could be affected by the angiotensinogen concentration and factors that influence the renin–renin substrate interaction, and displays suboptimal sensitivity in measuring low concentrations of renin [13]. PRC can be measured by chemiluminescence immunoassays, which circumvents many critical issues associated with radioimmunoassays, such as long incubation time, production of radioactive waste, the need to analyse several samples together to minimize the cost and employment of dedicated laboratory staff. However, a cross reaction between pro-renin, which circulates in significantly higher concentrations compared to the active enzyme, and renin could occur in immunoassays [14]. In the current case, both PRA and PRC are measured. However, the results are inconsistent; elevated PRC exists, while elevated PRA is absent in this patient, suggesting that PRC and subsequent SRVS, if PRC positive, should be incorporated in the differential diagnosis of secondary hypertension.

Aldosterone, as a mineralocorticoid hormone, can promote renal tubular sodium retention and potassium excretion, while excess aldosterone can cause hypertension, potassium wasting and hypokalaemia [13, 14]. Most reported cases of reninoma have hyperaldosteronism along with hypokalaemia [9, 13–15]. However, there can be an inconsistency between the existence of hyperaldosteronism and hypokalaemia. It has been reported that patients with PAC in the normal range could develop hypokalaemia [10]. In addition to promoting the secretion of aldosterone from the glomerular zone of the adrenal gland and then causing hypertension and hypokalaemia, angiotensin II (ATII), a downstream hormone of renin and another important member of the RAAS, can also cause an increase in blood pressure and hypokalaemia. This may explain why some patients can also present hypokalaemia even though they do not have hyperaldosteronism. In the current case, hypokalaemia was absent, secondary hyperaldosteronism was also absent, and hypertension was easily controlled. Although ATII was not measured, an elevated PAC with normal PRA suggests that an increased ATII may play a role in the clinical manifestation of this patient.

An interesting case reported that neither hypertension nor hypokalaemia was present in a patient with reninoma, suggesting that some reninoma may produce inactive renin that may be associated with nonsymptomatic or atypical reninoma [16]. Since hypertension and hypokalaemia are the joint effects of aldosterone and ATII, the clinical manifestations of patients depend on the degree of activation of the RAAS, including aldosterone and ATII. Indeed, the clinical presentation can vary from normal blood pressure with normal potassium levels to hypertension with normal potassium levels to severe and resistant hypertension with profound hypokalaemia [13, 14, 16]. It was therefore suggested that reninoma might be classified into three types based on clinical presentations: typical, atypical, and nonfunctioning [17].

**Fig. 3:**
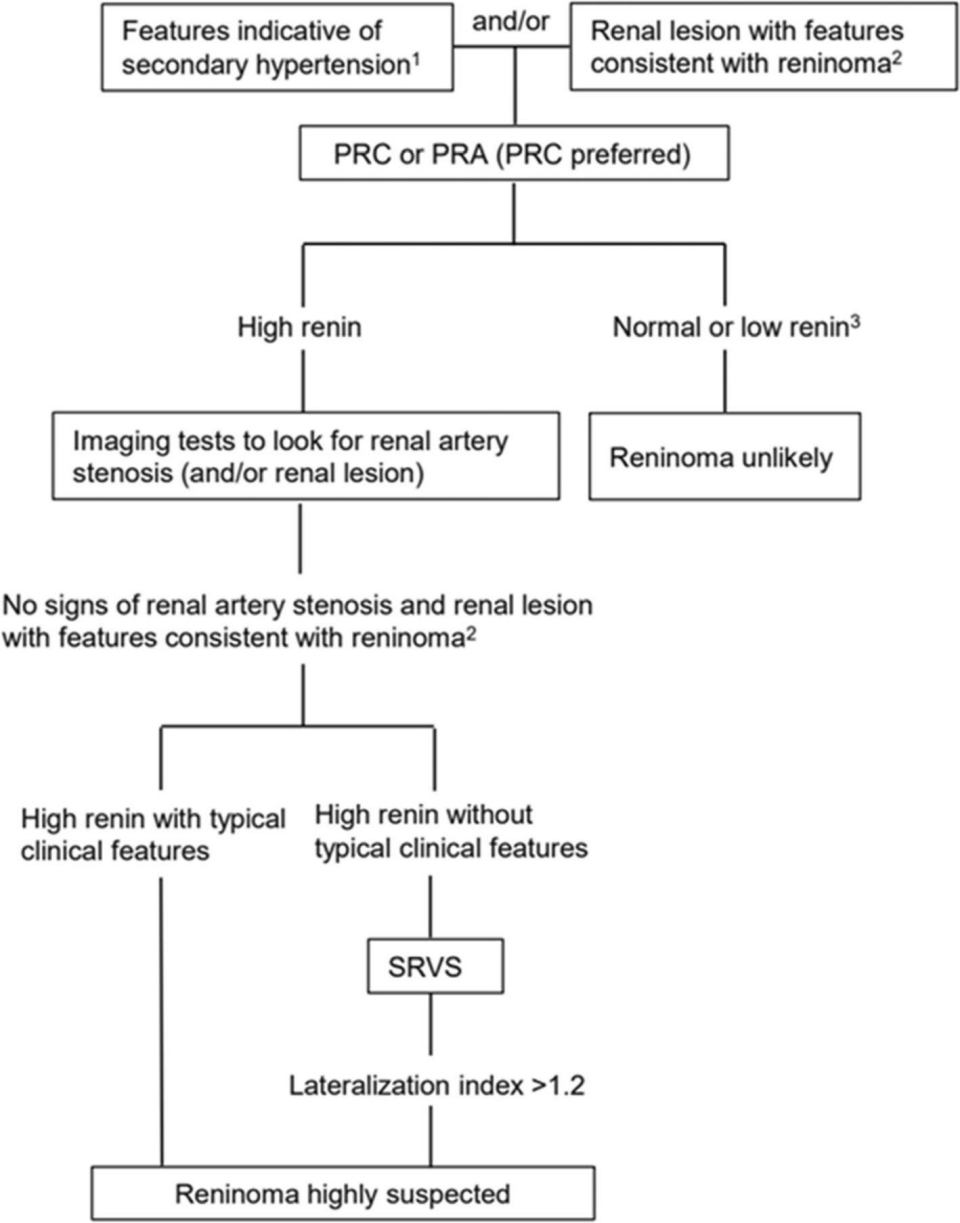
A proposed diagnostic flowchart for reninoma diagnosis. ^1^ Features indicative of secondary hypertension include young age at onset (less than 30), sudden onset of hypertension, uncontrolled refractory hypertension, malignant hypertension, and hypokalaemia. ^2^ Features of renal lesions indicative of reninoma include tumour occurring in adolescence or early adulthood, in female, small (2–3 cm) solitary subcapsular mass, hypo- to isodense to the renal parenchyma, hypovascular in the arterial phase, and delayed enhancement on CT scan, or T1 hypointense, T2 hypointense, and hypovascular after contrast administration on MRI. ^3^ If normal plasma renin activity, verify by measuring direct renin concentration. PRC, plasma renin concentration; PRA, plasma renin activity; SRVS, selective renal vein sampling

As a renal tumour, reninoma, especially for nonfunctioning and atypical reninoma, needs to be differentiated from other renal tumours, such as renal papillary carcinoma and renal clear cell carcinoma. MRI and ultrasound scanning only assist in localization of the tumour [9, 18], but they are of limited use in determining the type of the tumours [9, 19]. Currently, SRVS is increasingly used in the diagnosis of reninoma, and RVRR seems to be a reliable diagnostic tool for the lateralization of the tumour. It was reported that an RVRR of 1.2 gave a sensitivity of 85% and a specificity of 75%, whereas an RVRR of 1.5 maximized specificity while limiting sensitivity [3]. In the current case, an RVRR of 1.4 was diagnostic. In addition to RVRR, contrast-enhanced CT may be useful in the differential diagnosis. The typical presentation of reninoma was no obvious enhancement in the early arterial phase accompanied by delayed enhancement in the venous phase and extended scanning, but the degree of enhancement was weaker than that of the surrounding renal parenchyma, distinct from other types of renal tumours [17]. A proposed diagnostic flowchart for reninoma diagnosis is shown in Fig. 3. Evaluation of renin should be considered in patients with features indicative of secondary hypertension, i.e., young age at onset (less than 30), sudden onset of hypertension, uncontrolled refractory hypertension, malignant hypertension, hypokalaemia, or in patients with renal lesions indicative of reninoma, i.e., tumour occurs in adolescence or early adulthood, in female, small (2–3 cm) solitary subcapsular mass, hypo- to isodense to the renal parenchyma, hypovascular in the arterial phase, and delayed enhancement on CT scan, or T1 hypointense, T2 hypointense, and hypovascular after contrast administration on MRI [20–22]. To maximize the sensitivity for detecting reninoma, PRC is preferred over PRA in the evaluation of renin. If renin is low or normal, reninoma is unlikely; however, if the initial evaluation is PRA, PRC should be evaluated for verification. If high renin is detected, imaging tests should be performed to look for renal artery stenosis (and renal lesions). If a renal lesion with features indicative of reninoma is found and renal artery stenosis is excluded, reninoma is highly suspected in the context of typical clinical features. If typical clinical features are absent, selective renal vein sampling could be performed to further consolidate the diagnosis. A lateralization index >1.2 is indicative of reninoma.

Reninoma arises from the modified smooth muscle cells in the wall of the glomerular afferent arterioles that are part of the juxtaglomerular apparatus. In the current case, except for positive immunostaining for CD34 and vimentin, positive immunostaining for Syn was also found, consistent with previous reports [4, 10]. Immunohistochemical staining showed that the tumour cells had the characteristics of multidirectional differentiation and expressed epithelial, mesenchymal and neuroendocrine markers. Although some cases reported positive Syn immunostaining in the tumour cells, consistent with our current case, to date, there are no reports suggesting that reninoma could express SSTR2 and CgA.

This study has several limitations. First, the level of ATII was not measured. Therefore, there was no direct evidence supporting the effect of ATII in this patient. Second, owing to the lack of a prorenin assay in our hospital, plasma prorenin levels were not measured. Therefore, there was no direct evidence of elevated plasma inactive prorenin levels in this patient.

We report a unique case of reninoma that presented as mild hypertension without hyperaldosteronism and hypokalaemia, with normal PRA but elevated PRC, with lateralization confirmed in SRVS. This case highlights that PRC and subsequent SRVS, if PRC positive, should be incorporated into the differential diagnosis of secondary hypertension, especially in cases with renal masses suggestive of reninoma. The clinical features of reninoma may depend on the degree of activation of the RAAS. PRC rather than PRA may be a more sensitive marker for an atypical reninoma.

## Data Availability

The datasets analysed during the current study are available from the corresponding author on reasonable request.

## Abbreviations

ATII: Angiotensin II
ACTH: Adrenocorticotropic hormone
CgA: Chromogranin A
CT: Computed tomography
PRC: Plasma renin concentration
FDG: Fluorodeoxyglucose
MRI: Magnetic resonance imaging
PAC: Plasma aldosterone concentration
PET: Positron emission tomography
PRA: Plasma renin activity
RVRR: Renal vein renin ratio
SRVS: Selective renal vein sampling
Syn: Synaptophysin
SSTR2: Somatostatin receptor 2
